# Exacerbation of Psoriasis Following COVID-19 Vaccination: Report From a Single Center

**DOI:** 10.3389/fmed.2021.812010

**Published:** 2021-12-23

**Authors:** Yi-Wei Huang, Tsen-Fang Tsai

**Affiliations:** ^1^Department of Dermatology, National Taiwan University Hospital, Taipei, Taiwan; ^2^Department of Dermatology, College of Medicine, National Taiwan University, Taipei, Taiwan

**Keywords:** psoriasis, COVID-19, vaccine, exacerbation, HLA, human leukocyte antigen, Th17, biologics

## Abstract

The temporal association had been reported between vaccination and exacerbation of psoriasis, and episodes of psoriasis flare-up have recently been attributed to COVID-19 vaccines. We recruited 32 unimmunized controls and 51 vaccinated psoriasis patients, 49 of whom were under biological therapy, with regular clinic visits receiving a total of 63 shots of vaccines, including 30 doses of Moderna mRNA-1273 and 33 doses of AstraZeneca-Oxford AZD1222. Fifteen episodes of exacerbation attacked within 9.3 ± 4.3 days, which is higher than two episodes in the control group (*p* = 0.047). The mean post-vaccination severity of the worsening episodes increased from PASI 3.1 to 8.0 (*p* < 0.001). Three patients showed morphologic change from chronic plaque-type to guttate psoriasis. Deterioration of psoriasis following COVID-19 vaccination was not associated with age, sex, disease duration, psoriatic arthritis, family history of psoriasis, history of erythroderma, current biologics use, comorbidities, vaccine types, human leukocyte antigen (HLA)-C genotypes, baseline PASI nor pre-vaccination PASI. For those who received two doses of vaccination, all but one patient aggravated after the first shot but not the second. The mechanism of psoriasis exacerbation in immunized individuals is unclear, but Th17 cells induced by COVID-19 vaccines may play a role. In the pandemic era, psoriasis patients and physicians should acknowledge the possibility of fluctuation of disease activity when vaccinated against COVID-19. Nevertheless, compared to a treatable dermatologic disease with rapid resolution of exacerbation, psoriasis patients who do not have contraindications to vaccination should benefit from COVID-19 vaccines in the prevention of severe COVID-19 infection and fatality.

## Introduction

Psoriasis is a chronic immune-mediated cutaneous inflammatory disease that may be precipitated by drug, infection, stress, physical trauma, and vaccination (1–6). A lower rate of influenza vaccination in psoriasis patients may be attributed to the fact that vaccines may be a triggering factor for aggravation (7). “Psoriasis vaccinalis” had been described in different types of vaccines, including influenza, Bacillus Calmette-Guerin, tetanus-diphtheria, and pneumococcal polysaccharide vaccines (8). Patients may present as widespread severe psoriasis or new-onset guttate psoriasis. Recently, coronavirus (COVID-19) vaccinations have been linked to the exacerbation of psoriasis (9–11).

This study aims to evaluate the clinical characteristics and genetic factors in the aggravation of psoriasis after COVID-19 vaccination.

## Method

The study was approved by the Research Ethics Committee of National Taiwan University Hospital (201904124RINC). Consecutive patients with moderate to severe psoriasis who received COVID-19 vaccines in our dermatologic outpatient clinic between June 2021 and October 2021 were enrolled for analysis. Therapeutic inclusion criteria include patients under biologics and patients under remission after discontinuation of biologics, currently with/without traditional systemic treatment. The types of COVID-19 vaccine were documented, either Moderna mRNA-1273 or AstraZeneca-Oxford AZD1222. All patients were tested for human leukocyte antigen-C (HLA-C) genotypes. The baseline Psoriasis Area Severity Index (PASI) was defined as the most severe PASI before the initiation of current biological treatment, while the pre-vaccination PASI was defined as the PASI before receiving COVID-19 vaccines. Worsening of vaccinated patients was defined as (1) worsening of 50% PASI from a prior visit, which was based on an interval of 4–12 weeks depending on the biological agents, or (2) morphologic change, for example, chronic plaque-type to guttate, pustular or erythrodermic psoriasis, without other identifiable aggravating factors within 14 days of vaccination. Psoriasis area and severity index (PASI) was assessed at each clinic visit by the same physician. Aggravation of unvaccinated patients was defined as worsening 50% PASI compared to baseline PASI or morphological change. Possible precipitating factors, including upper respiratory tract infection, excess ultraviolet light exposure, alterations of medications, and psychological stress, are inquired orally.

Statistics analysis was performed using SPSS version 25. Parametric data are presented as mean ± SD. To compare intergroup differences, Shapiro-Wilk test was applied to determine the data normality of distribution. Based on the result, Mann-Whitney or Student's *t*-test was employed for quantitative variables. Pearson Chi-square test or Fisher's exact tests were applied for categorical data. The analysis results are two-tailed, with a significance level of 0.05.

## Results

A total of 83 patients were recruited, including 51 vaccinated patients receiving 63 doses of vaccines and 32 patients who did not receive COVID-19 vaccines (Table 1). COVID-19 vaccines include 30 doses of Moderna and 33 doses of AstraZeneca-Oxford. The age in the vaccinated group was 55.3 ± 11.6 years with a body weight of 78.0 ± 15.5 kg. Female patients comprise 27% (*n* = 14) of the vaccinated group. In the unvaccinated control, age was 50.4 ± 12.7 years, body weight was 71.6 ± 13.3, and female patients accounted for 44%. Age, sex, and body weight are not statistically different between the vaccinated and control group. All of the patients suffered from long-lasting psoriasis, with a mean disease duration of 18.0 ± 10.0 and 18.1 ± 9.6 years in the vaccinated and unvaccinated group, respectively. In patients who received COVID-19 vaccines, psoriatic arthritis was diagnosed in 61%, history of erythrodermic change was recorded in 16%, and positive family history was found in 31%. Among individuals not receiving COVID-19 vaccines, the percentages of psoriatic arthritis, history of erythroderma, and family history of psoriasis stood at 50, 29, and 22%, respectively, showing no difference when each was compared with the unvaccinated counterpart.

**Table 1:** Comparison between psoriasis patients vaccinated and unvaccinated against COVID-19.

	**Vaccinated**	**Unvaccinated**	***P*-value**
Number of patients, n	51	32	NA
Doses of vaccines, n	63	0	NA
Moderna mRNA-1273	30	0	NA
AstraZeneca-Oxford AZD1222	33	0	NA
Age (years), mean ± SD	55.3 ± 11.6	50.4 ± 12.7	0.077
Female, n (%)	14 (27%)	14 (44%)	0.155
Body weight (kg), mean ± SD	78.0 ± 15.5	71.6 ± 13.3	0.111
Disease duration (years), mean ± SD	18.0 ± 10.0	18.1 ± 9.6	0.771
Psoriatic arthritis, n (%)	31 (61%)	16 (50%)	0.370
History of erythroderma, n (%)	8 (16%)	9 (29%)	0.263
Family history of psoriasis, n (%)	15 (29%)	7 (22%)	0.610
Comorbidities			
Hypertension, n (%)	13 (26%)	7 (22%)	0.796
Diabetes mellitus, n (%)	9 (18%)	4 (13%)	0.758
Cardiovascular disease, n (%)	3 (6%)	0	0.281
Hepatitis B virus infection, n (%)	5 (10%)	4 (13%)	0.728
Hepatitis C virus infection, n (%)	2 (4%)	0	0.520
Numbers of exacerbation episodes, n (%)	15 (29%)	2 (6)	0.047
Interval between exacerbation and vaccine (days), mean ± SD	9.3 ± 4.1	NA	NA
Morphology change, n (%)	3 (5%)	0	0.548
HLA-C allele frequency (%)			
C*01	40.5	31.3	0.267
C*03	12.7	20.3	0.201
C*04	3.2	4.7	0.690
C*06	2.4	3.1	>0.999
C*07	26.2	23.4	0.727
C*08	4.8	6.3	0.735
C*12	4.0	4.7	>0.999
C*14	3.2	1.6	0.665
C*15	3.2	4.7	0.690
Current treatment			
Non-biologic only, n (%)	3 (6%)	1 (3%)	>0.999
Biologics, n (%)	48 (94%)	31 (97%)	>0.999

*HLA, human leukocyte antigen; NA, not applicable*.

The comorbidities include hypertension in 13 (26%), diabetes mellitus in 9 (18%), cardiovascular disease in 3 (6%), hepatitis B virus infection in 5 (10%), and hepatitis C virus infection in 2 (4%) vaccinated patients, whereas 7 (22%) have hypertension, 4 (13%) have diabetes mellitus, and 4 (13%) hepatitis B virus infection in the unvaccinated group. None of the patients in the control group have documented cardiovascular disease or hepatitis C virus infection.

**Figure 1:**
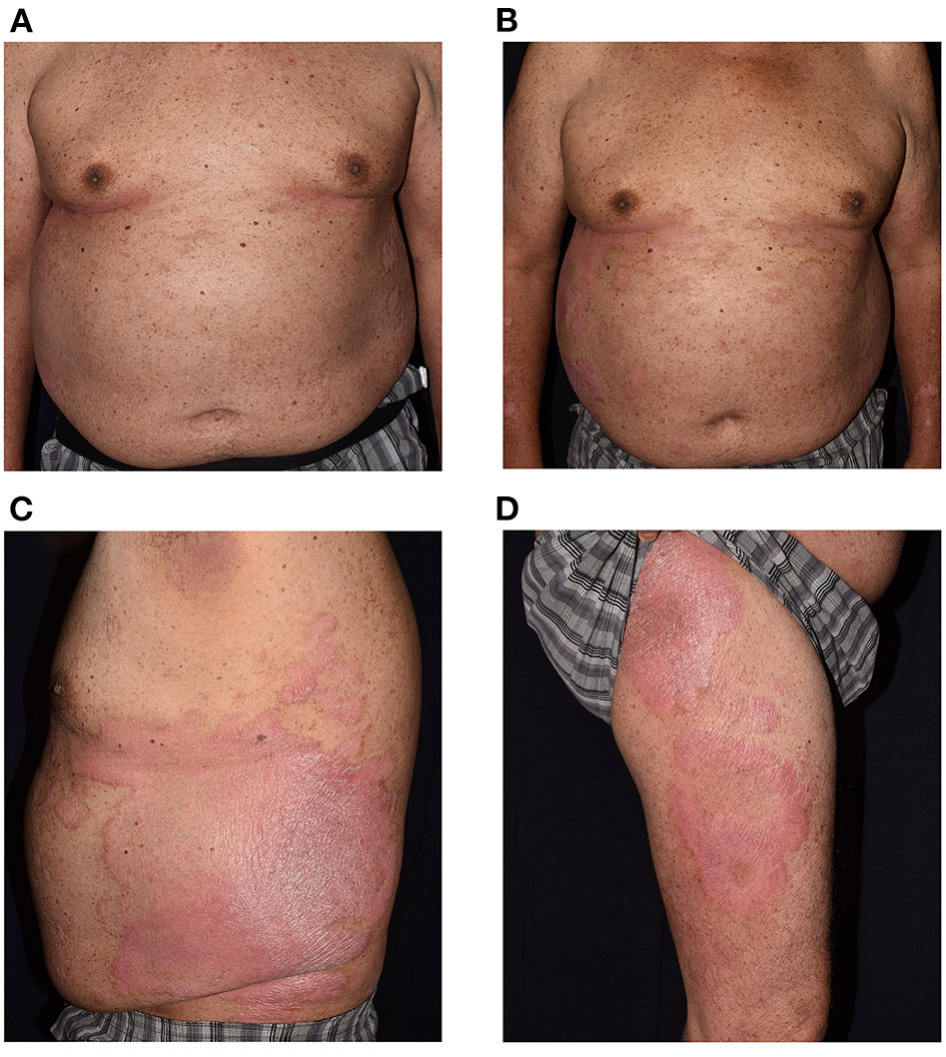
Clinical exacerbation of a 68-year-old man from baseline **(A)** Psoriasis Area Severity Index (PASI) at 5.4. Extensive erythematous scaly patches developed 14 days after Moderna vaccine, covering more than 13% of total body surface area, with PASI score at 10.0 **(B–D)**.

**Figure 2:**
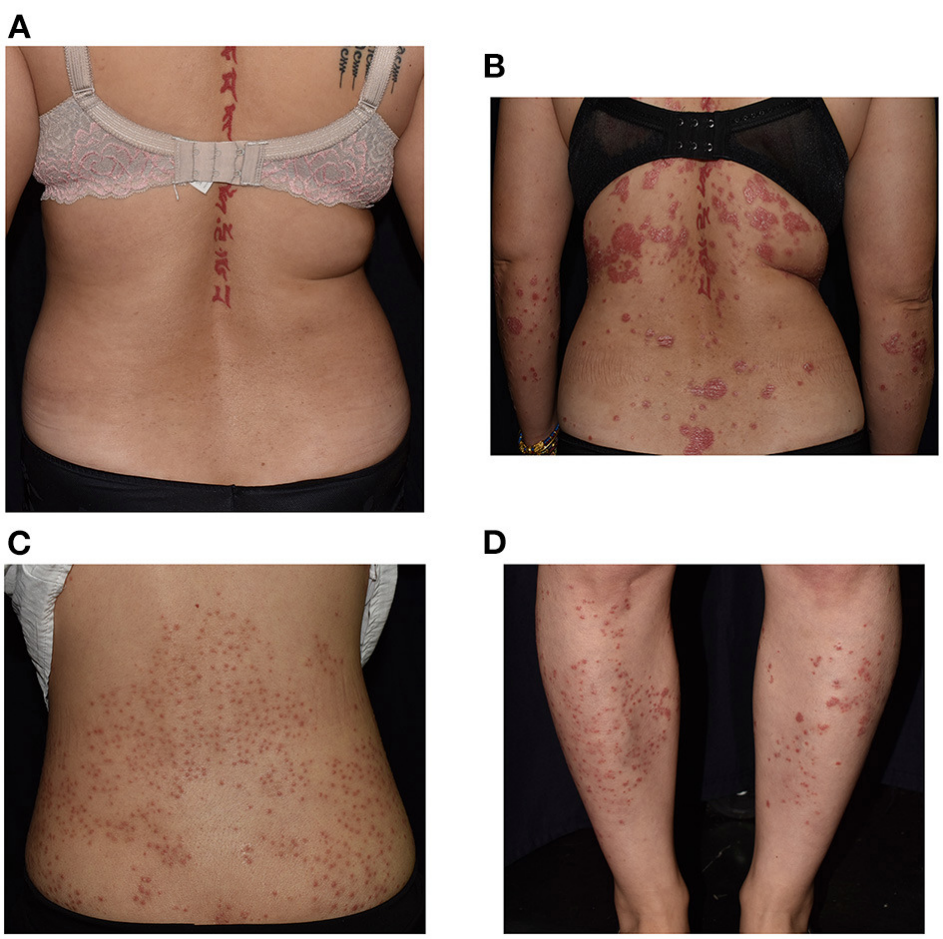
Severe exacerbation with morphological change in a 40-year-old woman with a history of psoriasis for more than a decade, worsening from Psoriasis Area Severity Index (PASI) 2.8 **(A)** to 10.7 **(B)**. Photos of the back **(C)** and lower legs **(D)** of a 39-year-old woman with chronic plaque-type psoriasis who developed guttate and/or follicular form 3 days after receiving AstraZeneca-Oxford vaccine.

Fifteen worsening episodes following administration of COVID-19 vaccine in psoriasis patients were observed (Figure 1), which is higher than two episodes recorded in the control group (*p* = 0.047). No specific aggravating factors, such as upper respiratory infection, excess ultraviolet exposure, change of medications, nor psychological stress, were reported in all patients. In the immunized group, three patients experienced morphologic changes from chronic plaque-type to guttate type (Figure 2).

The mean pre-vaccination PASI scores between those who deteriorated and the counterpart group are not significantly different (*p* = 0.571). The mean post-vaccination PASI of the worsening episodes significantly increased from 3.1 to 8.0 (*p* < 0.001), while the BSA increased from 2.4 to 8.0 (*p* = 0.061). In comparison, the mean post-vaccination PASI of the episodes not associated with exacerbation was stable over time (4.3–3.6, *p* = 0.329), and the BSA are not significantly different (3.5–2.6, *p* = 0.614).

The mean duration between vaccine injection to psoriasis deterioration was 9.3 ± 4.3 days. Among them, 11 showed improvement of disease severity in the following clinic visits, with an interval of 64.6 ± 29.7 days. As shown in Table 2, no specific HLA-C genotype is found to be related to worsening of skin manifestations. The result of the intergroup analysis is shown in Table 2. There was no difference between the exacerbation group and its counterpart regarding age, sex, disease duration, psoriatic arthritis, family history of psoriasis, history of erythrodermic psoriasis, current biologics use, comorbidities, nor the baseline PASI.

**Table 2:** Comparison between the exacerbation episodes and the exacerbation-free episodes in patients who received COVID-19 vaccines.

	**Exacerbation** **episodes**	**Exacerbation-free** **episodes**	***P*-value**
Female sex, n (%)	7 (46%)	11 (23%)	0.104
Age (years)	53.6 ± 12.2	55.5 ± 11.5	0.591
Vaccine type, AstraZeneca-Oxford/Moderna	8/7	25/23	>0.999
Disease duration (years)	20.1 ± 9.8	18.1 ± 10.3	0.378
Psoriatic arthritis, n (%)	7 (47%)	31 (65%)	0.241
Family history of psoriasis, n (%)	6 (40%)	15 (31%)	0.545
History of erythroderma, n (%)	3 (20%)	10 (21%)	>0.999
Baseline PASI	14.9 ± 8.8	12.5 ± 7.5	0.429
Pre-vaccination PASI	3.1 ± 1.8	4.3 ± 4.4	0.571
Current biologics use, n (%)	13 (87%)	47 (98%)	0.138
Comorbidities			
Hypertension, n (%)	5 (33%)	11 (23%)	0.501
Diabetes mellitus, n (%)	2 (13%)	7 (15%)	>0.999
Cardiovascular disease, n (%)	1 (7%)	3 (6%)	>0.999
Hepatitis B virus infection, n (%)	0	6 (13%)	0.321
Hepatitis C virus infection, n (%)	1 (7%)	2 (4%)	0.564
HLA-C allele frequency (%)			
C*01	38.5	46.7	0.523
C*03	11.5	16.7	0.531
C*04	3.1	3.3	>0.999
C*06	3.1	0	>0.999
C*07	27.1	23.3	0.814
C*08	6.3	0	0.334
C*12	4.2	3.3	>0.999
C*14	4.2	0	0.572
C*15	2.1	6.7	0.240

The same brands of vaccines were given to all the patients receiving two shots. A total of 12 patients received two doses of COVID-19 vaccination, including seven patients without aggravation, four patients showing exacerbation following the first injection but not the second one, and one patient repeatedly aggravated after vaccination, in whom AstraZeneca-Oxford was administered. In the subgroup of patients who only had worsening episodes once after the first dose of the COVID-19 vaccine, three of them received AstraZeneca-Oxford, and one received Moderna vaccine. Four and three patients were given AstraZeneca-Oxford and Moderna vaccines, respectively, in those whose disease severity was not worsened due to COVID-19 vaccines.

Regarding the treatment, only three patients were not receiving biologics; one was applying topical steroids, another taking methotrexate, and the other was taking acitretin. Forty-nine patients (94%) in the immunized group were under biological therapy, including guselkumab (*n* = 16), ixekizumab (*n* = 12), risankizumab (*n* = 6), etanercept (*n* = 4), adalimumab (*n* = 4), adalimumab plus methotrexate (*n* = 3), secukinumab (*n* = 2), and brodalumab (*n* = 1). In 14 individuals with disease aggravation, they are receiving guselkumab (*n* = 3), ixekizumab (*n* = 2), risankizumab (*n* = 2), etanercept (*n* = 1), adalimumab (*n* = 1), adalimumab plus methotrexate (*n* = 2), secukinumab (*n* = 1), methotrexate (*n* = 1), and topical steroid (*n* = 1). Whether receiving biological agents or not was not associated with disease exacerbation following COVID-19 vaccination (*p* = 0.138).

## Discussion

Reports of COVID-19 vaccines associated with psoriasis exacerbation were emerging (8, 9, 11). In an international registry of 414 individuals with cutaneous reactions after Pfizer-BioNTech and Moderna vaccines, two patients experienced psoriasis exacerbation (12). Besides worsening of pre-existing psoriatic lesions, a *de novo* generalized pustular psoriasis following administration of the first dose of AstraZeneca-Oxford COVID-19 vaccine was also reported (10). Recently, Ricardo et al. reported *de novo* nail psoriasis triggered by Pfizer-BioNTech in a 76-year-old woman (13).

Previously, psoriasis following Streptococcal infections is commonly reported, but its association with HLA-Cw6 is controversial (14). The relationship between genetic factors and vaccination in psoriasis aggravation has not been studied. However, widespread and unstable diseases were found in HLA-C positive patients (14). Whether worsening after COVID-19 vaccination results from the complex interplay between HLA and unstable disease remains to be clarified. A new insight provided by our report is that all patients received genetic testing for HLA-C. The relatively low HLA-Cw6 positivity in Chinese patients has been reported, especially in high need patients (biologic users) with moderate to severe psoriasis in which HLA-Cw1 was thought to play a more significant role (15). However, there was no significant association between a specific HLA-C allele and aggravation of disease activity after COVID-19 vaccination.

In our report, episodes of worsening of psoriasis were defined as 50% of deterioration of PASI scores, which is mainly based on the definition of minimal significant psoriasis efficacy endpoint (16) and relapse in clinical trials after discontinuation of biological agents, which is 50% of reduction of PASI improvement (17, 18). We suggest that changing clinical morphology should be regarded as a sign of disease exacerbation after receiving the COVID-19 vaccine. It is consistent with the definition of adverse events of trials of biologics for psoriasis. Three patients in our cohort developed guttate psoriasis even though all of them were diagnosed with chronic plaque-type psoriasis for more than a decade. More than hundreds of guttate lesions erupted four days after vaccination in one of the chronic plaque-type psoriasis patients.

The mean interval between COVID-19 vaccination and disease exacerbation was 9.3 days in our cohort, which was similar to another preliminary report from Greece (10.36 days) (8). In consistence with previous reports, no specific type of vaccine was associated with a significantly higher rate of exacerbation (8). In our cohort, there is one patient who showed exacerbation of psoriasis after both doses of COVID-19 vaccination. She is a 50-year-old woman receiving AstraZeneca-Oxford vaccines, showing surges of PASI scores 8 and 11 days after the first and second injections, respectively. Under regular ixekizumab administration, the disease severity was later controlled. The HLA-C serotyping showed she has HLA-Cw1/Cw10.

Psoriasis in four patients worsened after the first dose but not after the second. Three of them received AstraZeneca-Oxford vaccine, and one of them received Moderna vaccine. In addition to the possible triggering effect of COVID-19 vaccines, psoriasis severity may be altered by the effect of biologics, for example, time of initiation of the treatment course, duration of therapy, and the interval between COVID-19 vaccination and clinic visit. In our patients, two of them initiated guselkumab within 3 months before the first shot of COVID-19 vaccination. PASI response of patients receiving guselkumab increases with the duration of treatment (19). Another patient shifted from guselkumab to risankizumab after exacerbation following the first dose of Moderna. Since exacerbation are defined by physician-assessed PASI scores, mild attacks may occur between clinic visits but are not documented.

COVID-19 vaccination may be a triggering factor for psoriasis, as suggested by the short time intervals between vaccination and psoriasis exacerbation, which is supported by this and previous reports (8). Most of the currently used COVID-19 vaccines are based on adenovirus as vector or mRNA; thus, the immunologic reaction to the COVID-19 vaccine may be distinct from the influenza vaccine, which is mediated by T-helper (Th)1 and Th17 responses (7). Previous studies reported an increase in tumor necrosis factor (TNF)-α and interferon (IFN)-γ production by CD4+ T cells after AstraZeneca-Oxford COVID-19 vaccine (20). TNF-α is well-known as a potent proinflammatory cytokine in psoriatic skin lesions (21), whereas IFN-γ has been recognized as one of the pathogenic cytokines that can trigger inflammatory cascades of psoriasis with the potential to become a severity marker (22, 23). The critical role of the Th17 subset of CD4+ T cells, possibly IL-6-induced, in COVID-19 immunopathology and vaccine-induced immune enhancement was highlighted by recent studies (24–26). Interwoven with Th17, TNF-α, and IFN may be the link between psoriasis exacerbation and COVID-19 vaccines, yet further investigations are required to unravel the immunologic reactions. Further investigations and large controlled studies are warranted to elucidate the relationship between psoriasis and COVID-19 vaccines.

The limitations of the study are the small number of patients and possible fluctuation of disease course in patients with moderate to severe psoriasis. Although more patients under COVID-19 vaccination can be included, we included only patients who received severity assessment immediately before and after the vaccination. Besides, only patients with stable disease conditions for at least 3 months prior to vaccination without other identifiable aggravation factors were included. Although we only included psoriasis patients who aggravated in 2 weeks after vaccination to avoid recollection bias, this may result in over-estimation of the incidence of vaccine-induced psoriasis aggravation, based on the possibility that aggravation may urge the patients to seek medical attention before the scheduled visit. However, the proportion of patients with an unscheduled return to the clinic is low, at 6.3%.

Vaccination for COVID-19 is currently recommended for all patients with psoriasis, irrespective of the severity and current mediation, although temporary discontinuation may be needed for some oral systemic agents, but not biologics for psoriasis (27). This recommendation is based on the documented efficacy of the COVID-19 vaccine in the prevention of severe COVID-19 infection and fatality (28). In a large international series of patients with psoriasis and COVID-19 infection, 348 patients (93%) fully recovered from COVID-19, 77 (21%) were hospitalized, and 9 (2%) died (29). Patients under biological agents were associated with a lower risk of COVID-19-related hospitalization compared to those under systemic therapies (29). COVID-19 infection, rather than COVID-19 vaccine, can also exacerbate psoriasis (30, 31). Compared to a treatable dermatologic disease with rapid resolution of exacerbation, patients with psoriatic disease who do not have contraindications to vaccination should follow the guidance statements published by the National Psoriasis Foundation to receive an mRNA-based COVID-19 vaccine as soon as it becomes available to them (32).

In some patients, COVID-19 vaccinations may be associated with disease exacerbation of psoriasis, with an average interval of approximately 10 days. These abrupt clinical deteriorations are irrelevant to the type of vaccines injected, the baseline or pre-vaccination PASI, or the HLA-C genotyping. Psoriasis patients should be consulted before getting vaccinated for COVID, and prompt clinical visit should be available if exacerbation develop. However, more studies are needed to identify the true incidence and factors contributing to the aggravation.

## Data Availability Statement

The original contributions presented in the study are included in the article/supplementary material, further inquiries can be directed to the corresponding author/s.

## Ethics Statement

The studies involving human participants were reviewed and approved by Research Ethics Committee of National Taiwan University Hospital (201904124RINC). The patients/participants provided their written informed consent to participate in this study.

## Author Contributions

Y-WH and T-FT contributed to conception and design of the study. Y-WH organized the database, performed the statistical analysis, and wrote the first draft of the manuscript. All authors contributed to manuscript revision, read, and approved the submitted version.

## Conflict of Interest

T-FT has conducted clinical trials or received honoraria for serving as a consultant for Abbvie, Boehringer Ingelheim, Bristol-Myers Squibb, Celgene, Eli-Lilly, Galderma, Janssen-Cilag, Merck Sharp and Dohme, Novartis International AG, Pfizer Inc., and UCB Pharma. However, none of the above has direct conflict regarding this manuscript. The remaining author declares that the research was conducted in the absence of any commercial or financial relationships that could be construed as a potential conflict of interest.

## Publisher's Note

All claims expressed in this article are solely those of the authors and do not necessarily represent those of their affiliated organizations, or those of the publisher, the editors and the reviewers. Any product that may be evaluated in this article, or claim that may be made by its manufacturer, is not guaranteed or endorsed by the publisher.

## References

[1] Gunes AT Fetil E Akarsu S Ozbagcivan O Babayeva L. Possible triggering effect of influenza vaccination on psoriasis. J Immunol Res. (2015) 2015:258430. 10.1155/2015/258430 26380315 PMC4562095

[2] Kamiya K Kishimoto M Sugai J Komine M Ohtsuki M. Risk factors for the development of psoriasis. Int J Mol Sci. (2019) 20:4347. 10.3390/ijms20184347 31491865 PMC6769762

[3] Shi CR Nambudiri VE. Widespread psoriasis flare following influenza vaccination. Vaccine. (2017) 35:4785–6. 10.1016/j.vaccine.2017.06.067 28669619

[4] Shin MS Kim SJ Kim SH Kwak YG Park HJ. New onset guttate psoriasis following pandemic H1N1 influenza vaccination. Ann Dermatol. (2013) 25:489–92. 10.5021/ad.2013.25.4.489 24371399 PMC3870220

[5] Raaschou-Nielsen W. Psoriasis vaccinalis; report of two cases, one following B. CG vaccination and one following vaccination against influenza. Acta Dermato Venereol. (1955) 35:37–42. 14387477

[6] Yeh MH Tsai T-F. Clinical analysis of psoriatic inpatients-A 10-year retrospective study. Dermatol Sin. (2007) 25:103–11. 10.29784/DS.200706.0003

[7] Kromer C Wellmann P Siemer R Klein S Mohr J Pinter A. Influenza vaccination in psoriatic patients-epidemiology and patient perceptions: a german multicenter study (Vac-Pso). Vaccines (Basel). (2021) 9:843. 10.3390/vaccines9080843 34451968 PMC8402561

[8] Munguía-Calzada P Drake-Monfort M Armesto S Reguero-del Cura L López-Sundh AE González-López MA. Psoriasis flare after influenza vaccination in Covid-19 era: A report of four cases from a single center. Dermatol Ther. (2021) 34:e14684. 10.1111/dth.14684 33331024 PMC7883046

[9] Sotiriou E Tsentemeidou A Bakirtzi K Lallas A Ioannides D Vakirlis E. Psoriasis exacerbation after COVID-19 vaccination: a report of 14 cases from a single centre. J Eur Acad Dermatol Venereol. (2021) 35:e857–9. 10.1111/jdv.17582 34363647 PMC8447325

[10] Krajewski PK Matusiak Ł Szepietowski JC. Psoriasis flare-up associated with second dose of Pfizer-BioNTech BNT16B2b2 COVID-19 mRNA vaccine. J Eur Acad Dermatol Venereol. (2021) 35:e632–e4. 10.1111/jdv.17449 34131967 PMC8447171

[11] Chao J-P Tsai T-F. Psoriasis flare following ChAdOx1-S/nCoV-19 vaccination in patients with psoriasis under biologic treatment. Dermatol Sin. (2021). 10.4103/ds.ds_45_21. [Epub ahead of print].

[12] McMahon DE Amerson E Rosenbach M Lipoff JB Moustafa D Tyagi A. Cutaneous reactions reported after moderna and Pfizer COVID-19 vaccination: a registry-based study of 414 cases. J Am Acad Dermatol. (2021) 85:46–55. 10.1016/j.jaad.2021.03.092 33838206 PMC8024548

[13] Ricardo JW Lipner SR. Case of *de novo* nail psoriasis triggered by the second dose of Pfizer-BioNTech BNT162b2 COVID-19 messenger RNA vaccine. JAAD Case Rep. (2021) 17:18–20. 10.1016/j.jdcr.2021.09.009 34611542 PMC8480201

[14] Chen L Tsai T-F. HLA-Cw6 and psoriasis. Br J Dermatol. (2018) 178:854–62. 10.1111/bjd.16083 29072309

[15] Huang Y-W Tsai T-F. HLA-Cw1 and Psoriasis. Am J Clin Dermatol. (2021) 22:339–47. 10.1007/s40257-020-00585-1 33460021 PMC7812566

[16] Carlin CS Feldman SR Krueger JG Menter A Krueger GG. A 50% reduction in the Psoriasis Area and Severity Index (PASI 50) is a clinically significant endpoint in the assessment of psoriasis. J Am Acad Dermatol. (2004) 50:859–66. 10.1016/j.jaad.2003.09.014 15153885

[17] Wang X-Y Zhang C-L Wang W-H. Time to relapse after treatment withdrawal for different biologics used to treat plaque psoriasis. Chin Med J. (2020) 133:2998–3000. 10.1097/CM9.0000000000001232 33337760 PMC7752671

[18] Carey W Glazer S Gottlieb AB Lebwohl M Leonardi C Menter A. Relapse, rebound, and psoriasis adverse events: an advisory group report. J Am Acad Dermatol. (2006) 54(4 Suppl. 1):S171–81. 10.1016/j.jaad.2005.10.029 16488339

[19] Nakamura M Lee K Jeon C Sekhon S Afifi L Yan D. Guselkumab for the treatment of psoriasis: a review of phase III trials. Dermatol Ther (Heidelb). (2017) 7:281–92. 10.1007/s13555-017-0187-0 28639011 PMC5574739

[20] Ewer KJ Barrett JR Belij-Rammerstorfer S Sharpe H Makinson R Morter R. T cell and antibody responses induced by a single dose of ChAdOx1 nCoV-19 (AZD1222) vaccine in a phase 1/2 clinical trial. Nat Med. (2021) 27:270–8. 10.1038/s41591-020-01194-5 33335323

[21] Hawkes JE Chan TC Krueger JG. Psoriasis pathogenesis and the development of novel targeted immune therapies. J Allergy Clin Immunol. (2017) 140:645–53. 10.1016/j.jaci.2017.07.004 28887948 PMC5600287

[22] Johnson-Huang LM Suárez-Fariñas M Pierson KC Fuentes-Duculan J Cueto I Lentini T. A single intradermal injection of IFN-γ induces an inflammatory state in both non-lesional psoriatic and healthy skin. J Invest Dermatol. (2012) 132:1177–87. 10.1038/jid.2011.458 22277938 PMC3305841

[23] Abdallah MA Abdel-Hamid MF Kotb AM Mabrouk EA. Serum interferon-gamma is a psoriasis severity and prognostic marker. Cutis. (2009) 84:163–8. 19842576

[24] Hotez PJ Bottazzi ME Corry DB. The potential role of Th17 immune responses in coronavirus immunopathology and vaccine-induced immune enhancement. Microbes Infect. (2020) 22:165–7. 10.1016/j.micinf.2020.04.005 32305501 PMC7162764

[25] Xu Z Shi L Wang Y Zhang J Huang L Zhang C. Pathological findings of COVID-19 associated with acute respiratory distress syndrome. Lancet Respir Med. (2020) 8:420–2. 10.1016/S2213-2600(20)30076-X 32085846 PMC7164771

[26] Wu D Yang XO. TH17 responses in cytokine storm of COVID-19: an emerging target of JAK2 inhibitor Fedratinib. J Microbiol Immunol Infect. (2020) 53:368–70. 10.1016/j.jmii.2020.03.005 32205092 PMC7156211

[27] Curtis JR Johnson SR Anthony DD Arasaratnam RJ Baden LR Bass AR. American college of rheumatology guidance for COVID-19 vaccination in patients with rheumatic and musculoskeletal diseases: version 1. Arthritis Rheumatol. (2021) 73:1093–107. 10.1002/art.41734 33728796 PMC8250724

[28] Xing K Tu XY Liu M Liang ZW Chen JN Li JJ. Efficacy and safety of COVID-19 vaccines: a systematic review. Zhongguo Dang Dai Er Ke Za Zhi. (2021) 23:221–8. 10.7499/j.issn.1008-8830.2101133 33691913 PMC7969187

[29] Mahil SK Dand N Mason KJ Yiu ZZN Tsakok T Meynell F. Factors associated with adverse COVID-19 outcomes in patients with psoriasis-insights from a global registry-based study. J Allergy Clin Immunol. (2021) 147:60–71. 10.1016/j.jaci.2020.10.007 33075408 PMC7566694

[30] Kutlu Ö Metin A. A case of exacerbation of psoriasis after oseltamivir and hydroxychloroquine in a patient with COVID-19: will cases of psoriasis increase after COVID-19 pandemic? Dermatol Ther. (2020) 33:e13383. 10.1111/dth.13383 32259878 PMC7235511

[31] Ozaras R Berk A Ucar DH Duman H Kaya F Mutlu H. Covid-19 and exacerbation of psoriasis. Dermatol Ther. (2020) 33:e13632. 10.1111/dth.13632 32436303 PMC7280710

[32] Gelfand JM Armstrong AW Bell S Anesi GL Blauvelt A Calabrese C. National Psoriasis Foundation COVID-19 Task Force guidance for management of psoriatic disease during the pandemic: version 2-Advances in psoriatic disease management, COVID-19 vaccines, and COVID-19 treatments. J Am Acad Dermatol. (2021) 84:1254–68. 10.1016/j.jaad.2020.12.058 33422626 PMC7788316

